# Zinc Oxide-Loaded Recycled PET Nanofibers for Applications in Healthcare and Biomedical Devices

**DOI:** 10.3390/polym17010045

**Published:** 2024-12-28

**Authors:** Andreea Mihaela Grămadă (Pintilie), Alexandra-Elena Stoica (Oprea), Adelina-Gabriela Niculescu, Alexandra Cătălina Bîrcă, Bogdan Ștefan Vasile, Alina Maria Holban, Teodora Mihaiescu, Andreea Iren Șerban, Alina Ciceu, Cornel Balta, Simona Dumitra, Monica Puticiu, Florin Iordache, Anca Hermenean, Adina Alberts, Alexandru Mihai Grumezescu, Ovidiu Cristian Oprea, Simona Ardelean

**Affiliations:** 1 Department of Science and Engineering of Oxide Materials and Nanomaterials, National University of Science and Technology POLITEHNICA Bucharest, 011061 Bucharest, Romaniaadelina.niculescu@upb.ro (A.-G.N.); alexandra.birca@upb.ro (A.C.B.); agrumezescu@upb.ro (A.M.G.); 2 Research Institute of the University of Bucharest—ICUB, University of Bucharest, 050657 Bucharest, Romania; alina.m.holban@bio.unibuc.ro; 3 Research Center for Advanced Materials, Products and Processes, National University of Science and Technology POLITEHNICA Bucharest, 060042 Bucharest, Romania; bogdan.vasile@upb.ro; 4 National Research Center for Micro and Nanomaterials, National University of Science and Technology POLITEHNICA Bucharest, 060042 Bucharest, Romania; 5 Faculty of Biology, University of Bucharest, 030018 Bucharest, Romania; 6 Department of Preclinic Sciences, Faculty of Veterinary Medicine, University of Agronomic Sciences and Veterinary Medicine of Bucharest, 050097 Bucharest, Romania; teomihro@yahoo.co.uk (T.M.); andreea-iren.serban@fmvb.usamv.ro (A.I.Ș.); florin.iordache@fmvb.usamv.ro (F.I.); 7 “Aurel Ardelean” Institute of Life Sciences, Vasile Goldis Western University of Arad, 310414 Arad, Romania; ciceu.alina@uvvg.ro (A.C.); balta.cornel@uvvg.ro (C.B.); hermenean.anca@uvvg.ro (A.H.); 8 Faculty of Medicine, Vasile Goldis Western University of Arad, 310025 Arad, Romania; dumitra.simona@uvvg.ro (S.D.); puticiu.monica@uvvg.ro (M.P.); 9 Carol Davila University of Medicine and Pharmacy, 050474 Bucharest, Romania; 10 Department of Inorganic Chemistry, National University of Science and Technology POLITEHNICA Bucharest, 011061 Bucharest, Romania; ovidiu.oprea@upb.ro; 11 Faculty of Pharmacy, Vasile Goldis Western University of Arad, 310130 Arad, Romania; ardelean.simona@uvvg.ro

**Keywords:** polyethylene terephthalate nanofibers, zinc oxide nanoparticles, electrospun membranes, recycling, antimicrobial activity, biocompatibility

## Abstract

Polyethylene terephthalate (PET) is a widely utilized synthetic polymer, favored in various applications for its desirable physicochemical characteristics and widespread accessibility. However, its extensive utilization, coupled with improper waste disposal, has led to the alarming pollution of the environment. Thus, recycling PET products is essential for diminishing global pollution and turning waste into meaningful materials. Therefore, this study proposes the fabrication of electrospun membranes made of recycled PET nanofibers as a cost-effective valorization method for PET waste. ZnO nanoparticles were coated onto polymeric materials to enhance the antimicrobial properties of the PET fibers. Morphostructural investigations revealed the formation of fibrillar membranes made of unordered nanofibers (i.e., 40–100 nm in diameter), on the surface of which zinc oxide nanoparticles of 10–20 nm were attached. PET@ZnO membranes demonstrated effective antimicrobial and antibiofilm activity against Gram-positive and Gram-negative bacteria, yeasts, and molds, while imparting no toxicity to amniotic fluid stem cells. In vivo tests confirmed the materials’ biocompatibility, as no side effects were observed in mice following membrane implantation. Altogether, these findings highlight the potential of integrating ZnO nanoparticles into recycled PET to develop multifunctional materials suitable for healthcare facilities (such as antimicrobial textiles) and biomedical devices, including applications such as textiles, meshes, and sutures.

## 1. Introduction

Polyethylene terephthalate (PET) is one of the most utilized polymers in many industries, recognized for its remarkable physicochemical attributes, including lightweight, flexibility, durability, transparency, and resistance to moisture. These attributes have been especially valuable for the packaging sector, leading to PET usage for fabricating various bottles, food containers, and synthetic fibers [1,2,3]. However, with its substantial use in modern packaging solutions and the lack of responsibility for managing plastic waste, PET has become a major pollution threat [4,5]. PET poses significant environmental challenges mainly due to its persistence in the environment, affecting both marine and terrestrial ecosystems. Despite its durability being considered an advantage for the end product, inappropriate PET package littering in natural environments results in long degradation times and the accumulation of harmful microplastics [6,7,8,9].

Therefore, strategies have been implemented to mitigate the environmental impact of PET, and several mechanical and chemical recycling methods have been developed. As it only employs physical processes (e.g., grinding and remelting), mechanical recycling is the most common approach to managing PET waste. Nonetheless, chemical recycling techniques aimed at breaking the polymer into its monomers for recondensation provide a more suitable alternative for generating high-quality PET that can be used once more for varied applications [3,10]. The use of recycled PET not only significantly reduces the environmental impact but also helps conserve valuable resources and achieve sustainability goals while maintaining material performance. Therefore, advancing and diversifying recycling technologies is vital for boosting the efficiency and productivity of PET recycling processes. This further supports circular economy initiatives and reduces the reliance on virgin raw materials [11,12,13].

One promising method for processing recycled PET is electrospinning this material into fibers that can further generate new valuable products [14,15]. The electrospinning technique is a simple yet versatile and reliable fabrication process that offers control over fiber diameter and morphology to obtain fibers with precise structural characteristics suitable for developing advanced materials. Specifically, electrospun fibers can be further utilized in various applications, including wound dressings, scaffolds for tissue engineering, medical diagnosis devices, smart packaging, membranes, filters, and decontamination systems [16,17,18,19,20].

Such PET fibers benefit from low production costs, high tensile and impact strength, chemical resistance, processability, excellent flow properties, non-absorbability, and durability in physiological conditions, features that make them particularly appealing for biomedical applications [20,21,22,23]. Moreover, studies have shown that PET-based materials exhibit enhanced biocompatibility, interacting favorably with biological systems, minimizing inflammatory responses, and promoting cell adhesion and growth [20,24].

However, the porosity of this abiotic material makes it susceptible to microbial adhesion, further leading to bacterial colonization at the implantation site [25,26,27,28]. Recent studies have highlighted the importance of material interactions with biological systems, emphasizing both their safety and metabolic impact on the body [28,29,30,31], aspects relevant for the functionalization of PET in biomedical applications. Therefore, it is advisable to modify the material to mitigate the risk of microorganism contamination and reduce unwanted complications. Several approaches are considered to improve PET-based materials’ in vivo behavior, such as functionalization, grafting, coating, adding antimicrobials, or incorporating PET in polymer mixtures with enhanced properties [23,25,32,33,34]. The most common method for imparting antimicrobial properties to materials involves incorporating or embedding antimicrobial agents, such as antibiotics, cyclodextrins, metals, or metal-oxide nanoparticles, within nanofibers [14].

Among the wide range of potential metal-oxides, zinc oxide (ZnO) is recognized for its antimicrobial properties and biocompatibility, being certified as a safe food additive by the U.S. Food and Drug Administration (FDA) [35,36]. When moving from bulk metal oxide to its nanoparticulate form, the antimicrobial activity is even higher as the small particle dimensions are correlated with the size of biological systems they interact with, allowing enhanced intracellular uptake. Furthermore, the high surface-to-volume ratio of ZnO nanoparticles results in elevated surface energy and an enhanced capacity to produce reactive oxygen species (ROS), which can compromise microbial cell membranes [35,37,38,39,40,41,42,43,44].

These antimicrobial properties indicate the promising effects of incorporating ZnO nanoparticles in various advanced materials to inhibit microbial growth and improve product safety and longevity. Specifically, integrating ZnO nanoparticles into PET nanofibers can lead to multifunctional materials with enhanced mechanical strength and biocompatibility, making them highly beneficial for use in a wide array of applications, including enhanced medical devices [44,45,46,47].

In this context, this study aimed to synthesize and characterize electrospun recycled PET nanofibers loaded with ZnO nanoparticles, mainly focusing on establishing their physicochemical features, in vitro and in vivo biocompatibility, and antimicrobial properties. Although previous studies have explored PET nanofibers functionalized with ZnO for antimicrobial applications [47,48,49], our research provides a novel contribution by investigating the antimicrobial efficacy, biocompatibility, and long-term durability of these membranes. By demonstrating the significant enhancement of antibacterial performance and tissue regeneration potential, we offer a comprehensive analysis that is critical for future healthcare applications. Thus, our study’s novelty resides in identifying optimal conditions that maximize both microbial inhibition and cell proliferation, positioning PET@ZnO membranes as strong candidates for biomedical applications.

## 2. Materials and Methods

### 2.1. Materials

All chemical reagents utilized in this study were of analytical grade and procured from Sigma-Aldrich Merck (Darmstadt, Germany). They were used without additional purification to maintain consistency and reliability throughout the experimental procedures. For antimicrobial testing, microbial strains and growth conditions included Staphylococcus aureus ATCC 6538, Pseudomonas aeruginosa ATCC 27853, Candida albicans ATCC 10231, and Aspergillus niger ATCC 16888, all obtained from the American Type Culture Collection (ATCC, Manassas, VA, USA). Glycerol stocks were streaked on nutritive agar for bacteria, YPG (yeast peptone glucose) for yeast, and PDA (potato dextrose agar) for molds, ensuring fresh cultures for subsequent studies.

### 2.2. Fabrication of Nanostructured Membranes from Recycled PET

The electrospinning technique was utilized to transform recycled PET into nanostructured membranes. This method facilitated the fabrication of membranes featuring a fibrous network with interconnected, overlapping, and randomly oriented fibers.

Membranes based on recycled PET (used as controls in experiments) were prepared by dissolving 1 g of PET sourced from a carbonated juice package in a solution of 20 mL methylene chloride and 2 mL trifluoroacetic acid. The solution was homogenized using a magnetic stirrer.

A 4.54% PET solution was deposited onto an aluminum substrate using the needle electrospinning device (Tong Li Tech, Shenzhen, China) equipment, with a needle diameter of 21G (0.8 mm internal diameter). For the experiments, a 200 mm needle-to-target distance was maintained, and a voltage of 23.26 kV was applied (−5.73 kV (needle—Output1) and 17.53 kV (collector—Output2)). A heating system (0.6 kW) was used to maintain solution viscosity, and the process was carried out at a humidity of 35% and a temperature of 27 °C. The experiment was conducted at four different deposition rates: 10 mL/h, 7.5 mL/h, 5 mL/h, and 2.5 mL/h [14,15].

The samples obtained through the electrospinning deposition were removed from the aluminum substrate surface and sectioned into fragments measuring 1 cm × 1 cm.

The recycled PET-based membranes were subsequently used as substrates for the deposition of ZnO nanoparticles, yielding four types of PET@ZnO functionalized membranes (i.e., 10 mL/h, 7.5 mL/h, 5 mL/h, and 2.5 mL/h), according to the deposition rates.

### 2.3. Fabrication of PET-Based Membranes with ZnO Nanoparticles

In this study, we developed a method to prepare PET-based membranes embedded with ZnO nanoparticles using a dual-solution immersion process. The synthesis involved the preparation of two separate solutions. Solution 1 consisted of zinc nitrate, specifically 3 g of Zn(NO_3_)_2_·6H_2_O dissolved in 97 mL of methyl alcohol, which serves as a precursor for ZnO formation. Solution 2 was composed of sodium hydroxide, with 3 g of NaOH dissolved in 97 mL of water, used to facilitate the nanoparticle deposition onto the PET substrate. 

Before immersion in Solution 2, the PET membranes were pretreated by thorough cleaning with ethanol and distilled water to eliminate surface contaminants and residues that could interfere with the deposition process.

The PET sections were first immersed in Solution 2 and subjected to magnetic stirring for 10 min at room temperature (~25 °C). This immersion step activates the PET surface by introducing hydroxyl (–OH) groups [50], enhancing its hydrophilicity and promoting effective interaction with Zn^2^⁺ ions in the subsequent step. NaOH provides OH⁻ ions that react with Zn^2^⁺ to form Zn(OH)_2_, which then dehydrates to ZnO [51]. The bonding between the PET surface and ZnO involves multiple interactions, primarily hydrogen bonding between the functional groups on PET (such as C=O and –OH) and the ZnO nanoparticles. Additionally, electrostatic interactions occur between the negatively charged PET surface (due to deprotonated hydroxyl or carboxyl groups) and the positively charged Zn^2^⁺ ions during the deposition process.

Following this, the sections were transferred to Solution 1 for another 10 min under similar conditions. During this step, Zn^2^⁺ ions from Zn(NO₃)_2_ interact with the residual OH⁻ ions on the PET surface to form Zn(OH)_2_, which dehydrates into ZnO upon drying. After treatment, the membranes were removed from the solutions and allowed to air dry at room temperature overnight, setting the stage for subsequent characterization and evaluation of their properties. This method highlights a straightforward approach to enhancing the functionality of PET membranes with nanoparticle incorporation, potentially offering improved antimicrobial properties and utility in various industrial applications.

### 2.4. Characterization Methods

Fourier-Transform Infrared (FT-IR) Spectroscopy

Infrared spectra were recorded using a Nicolet iN10 MX FT-IR spectrometer (ThermoFisher Scientific, Waltham, MA, USA) over a wavenumber range of 4000–300 cm^−1^. Measurements were performed in reflection mode with a resolution of 4 cm^−1^. For each sample, 32 scans were acquired, co-added, and processed into absorbance data using the OmnicPicta software (Thermo Scientific, Waltham, MA, USA).

Scanning Electron Microscopy (SEM)

Samples were examined using a scanning electron microscope (FEI Company, Hillsboro, OR, USA) to analyze the morphology and size of the nanostructured membranes. Micrographs were captured by detecting secondary electron signals at an energy of 30 keV.

Transmission Electron Microscopy (TEM)

For TEM imaging, samples were deposited onto a carbon-coated copper grid at ambient temperature. High-resolution TEM micrographs were acquired using a Tecnai™ G2 F30 S-TWIN microscope (FEI Company, Hillsboro, OR, USA), operated in transmission mode at 300 kV. The microscope provided point and line resolution values of 2 Å and 1 Å, respectively.

X-ray Diffraction

Grazing incidence X-ray diffraction (GIXRD) was conducted using a Panalytical Empyrean diffractometer (PANalytical, Almelo, The Netherlands) with CuKα radiation (wavelength: 1.541874 Å). The system was equipped with a 2 × Ge (220) hybrid monochromator for Cu and a parallel plate collimator on the PIXcel3D detector. The scanning was performed along the 2θ axis over a range of 5° to 80°, with an incidence angle of 0.5°, a step size of 0.04°, and a time per step of 3 s.

Thermogravimetric Analysis

The thermal analyses were conducted using a Shimadzu DTG-TA-50H instrument (Carlsbad, CA, USA) over a temperature range from room temperature to 900 °C, with a heating rate of 10 K · min^−1^. All measurements were carried out under a flow of dried synthetic air (80% N_2_ and 20% O_2_) at a purge rate of 20 mL · min⁻^1^.

In vitro biocompatibility

To test the biological effects (i.e., cytotoxicity and antibacterial efficiency) of the obtained PET materials, specimens were cut to 1 cm × 1 cm dimensions and sterilized by UV radiation exposure for 30 min on each side.

(a) Cytotoxicity assessment

The cytotoxicity of the PET-based membranes was evaluated using human diploid cells, which serve as a reliable model for assessing biocompatibility in medical applications. These cells were selected due to their relevance in mimicking the behavior of human tissues, allowing us to observe potential effects on cell proliferation and the overall compatibility of the material for healthcare use [52].

In more detail, an MTT assay was performed using the Vybrant MTT Cell Proliferation Assay Kit (Molecular Probes, Eugene, OR, USA). Amniotic fluid stem cells (AFSCs) were cultured in 96-well plates at a seeding density of 3000 cells/well under various experimental conditions (in the presence of the evaluated PET-based samples). Subsequently, 10 µL of 12 mM MTT was added and incubated at 37 °C for 4 h. Then, 100 µL of SDS-HCl solution was added, and the plate was pipetted vigorously to solubilize the formazan crystals. After 1 h of incubation, the mixture was pipetted for homogenization and to eliminate bubbles that could interfere with the reading. The absorbance was measured at 570 nm using a spectrophotometer (TECAN, Männedorf, Switzerland) [14,15].

(b) Oxidative stress assessment

Amniotic fluid-derived mesenchymal stem cells (AFSCs) were seeded at a density of 3000 cells per well in 96-well plates containing 300 µL of DMEM culture medium supplemented with 10% fetal bovine serum and 1% antibiotics (penicillin, streptomycin/neomycin). After 24 h of incubation, the cells were exposed to biomaterials for subsequent analyses. Oxidative stress was evaluated using the GSH-Glo™ Glutathione Assay kit (Promega, Madison, WI, USA), which measures levels of reduced glutathione (GSH), a key antioxidant molecule. This assay utilizes a luciferin derivative substrate (Luciferin-NT), which is enzymatically converted to luciferin in the presence of GSH, a reaction catalyzed by glutathione S-transferase (GST) included in the kit. The luciferin produced in the first reaction is detected as a luminescent signal generated by the luciferase enzyme in a second coupled reaction. The luminescent signal generated is proportional to the amount of GSH present in the sample. For the assay, the culture medium is removed, and 100 µL of 1X GSH-Glo Reagent (comprising Luciferin-NT substrate and GST diluted 1:100 in GSH-Glo Reaction Buffer) is added to each well. After 30 min of incubation at 37 °C, 100 μL of Luciferin Detection Reagent is added, followed by another 15 min of incubation at 37 °C. After this, the cell culture medium is well mixed, and the plate is read using a luminometer. To facilitate the conversion of luminescence (in RLU) to GSH concentration, a GSH standard curve ranging from 0 to 5 µM was prepared [14,15].

Antimicrobial effect

(a) The effect of PET-based membranes on planktonic microorganisms’ growth

To test the effect of the PET-based membranes, the samples were cut to 1 cm × 1 cm dimensions and sterilized by UV radiation exposure for 20 min on each side. UV-sterilized PET-based membranes were used to evaluate their antibacterial effects against free-floating (planktonic) microbial cultures. In each well of a sterile 6-well plate, an individual PET-based membrane fragment was placed, covered with 2 mL of liquid medium (nutritive broth for bacteria and YPG broth for yeasts), and 20 μL of microbial suspension at a density of 0.5 McFarland for bacteria or 1 McFarland for yeasts, prepared in sterile 0.9% NaCl solution. After 24 h of incubation at 37 °C, 200 µL of the microbial suspensions were transferred to a sterile 96-well plate, and the turbidity of the microbial cultures was measured spectrophotometrically at 600 nm.

(b) Evaluation of Adhesion and Biofilm Formation

An individual sterile material fragment was placed in each well of a 6-well sterile plate. Subsequently, 2 mL of liquid growth medium was added to each well, followed by inoculation with 20 μL of a microbial suspension prepared in sterile physiological saline (0.9% NaCl) at a density of 0.5 McFarland or 1 McFarland for yeasts. The plates were then incubated at 37 °C for 24 h. Following this initial incubation, the materials were rinsed with sterile saline, and the medium was changed to allow for the development of biofilms. The plates were incubated for various time periods (24, 48, and 72 h) to demonstrate the microbial capacity for adhesion and biofilm formation. After each incubation interval considered, the PET-based membrane fragments with the developed biofilm were washed with sterile saline and transferred to sterile tubes containing 1 mL of sterile saline. Biofilm cells were detached by vortexing the tubes vigorously for 30 s, followed by 10 s of sonication. The resulting cell suspensions were serially diluted, and aliquots of these dilutions were plated onto solid culture media to quantify the number of colony-forming units (CFUs).

(c) Assessment of antifungal (mold) effect

On the surface of YPG agar distributed in Petri dishes (Ø = 10 cm), an inoculum consisting of a spore suspension of *Aspergillus niger* in sterile saline solution supplemented with 1% Tween 80 was streaked. In the center of each Petri dish inoculated in this way, a fragment of the obtained PET-based membranes (dimensions: 1 cm × 1 cm, previously sterilized) was added, and the plates were incubated at room temperature for 4 weeks, with weekly inspections to observe the appearance of the test samples in the presence of fungal cultures.

In Vivo Experimental Design

The in vivo study was conducted using CD1 mice provided by the Animal Facility of Vasile Goldis Western University of Arad. The experimental protocol was reviewed and approved by the University’s Research Ethics Commission. CD1 mice were housed in cages with regulated airflow and maintained on a standard 12-h light/dark cycle. Pre-sterilized materials (UV for 30 min) were implanted subcutaneously in the dorsal region under intraperitoneal anesthesia of 100 mg/kg b.w. Ketamine hydrochloride and 10 mg/kg b.w. Xylazine hydrochloride. Mice were randomly assigned to five experimental groups (n = 20 each): control animals with skin incision but without an implant, a group of mice implanted with 2.5 mL/h PET, a group of mice implanted with 10 mL/h PET, a group of mice implanted with 2.5 mL/h PET@ZnO, and a group of mice implanted with 10 mL/h PET@ZnO. Within each group, half of the mice were euthanized at 24 h post-surgery and the remaining half at 7 days post-surgery. Following implantation, mice were housed individually. A veterinarian conducted daily clinical examinations, assessing the surgical incision for appearance, redness, infection, edema, abscess, hematoma, and scarring. Biopsies were taken at 24 h and 7 days post-surgery under anesthesia [14,15].

Histology

Biopsies, including the material and surrounding tissues, were fixed in 4% paraformaldehyde, subsequently embedded in paraffin, sectioned at 5 μm, and stained using hematoxylin and eosin (H&E) and Masson Goldner trichrome techniques. Microscopic examination was carried out with an Olympus BX43 microscope equipped with an Olympus XC30 digital camera and CellSens software. Histological sections were scored based on the degree of inflammatory infiltrate, fibroblast proliferation, and neovascularization, with each parameter evaluated on a scale of 0–4, from - (not present) to ++++ (extensive), according to our previous methodology [14].

## 3. Results

### 3.1. Physicochemical Characterization

**Figure 1:**
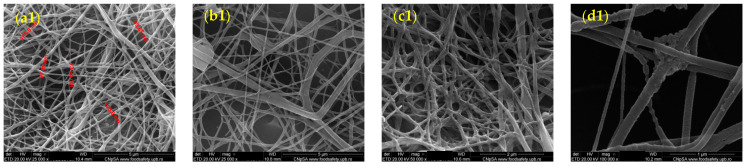

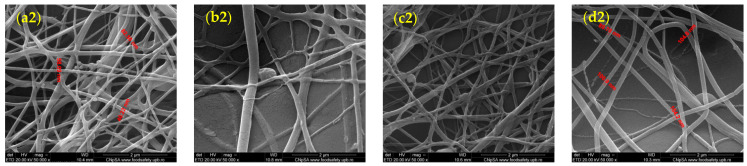
SEM images recorded for PET@ZnO at various deposition rates, where (**a1**,**2**)—2.5 mL/h; (**b1**,**2**)—5 mL/h; (**c1**,**2**)—7.5 mL/h; (**d1**,**2**)—10 mL/h.

The nanostructured membranes based on PET, coated with ZnO, were characterized using SEM. The results obtained for the four different deposition rates used to produce the nanostructured membranes are presented in Figure 1. A network of unordered fibers is observed, on the surface of which non-uniformly distributed granular nanostructures are evident, with sizes ranging between 10 and 20 nm. Additionally, it is noted that the fibers have diameters varying between 40 and 100 nm.

**Figure 2:**
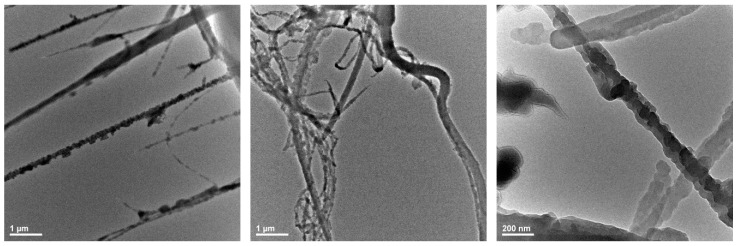
TEM images recorded for the nanostructured PET@ZnO membranes, with a deposition rate of 2.5 mL/h.

The experimental variants obtained were also characterized using TEM. The results for the deposition rate of 2.5 mL/h are presented in Figure 2. The analysis of the recorded images reveals the fibrous structure of the membrane and granules on the fiber surfaces, confirmed in size by SEM.

**Figure 3:**
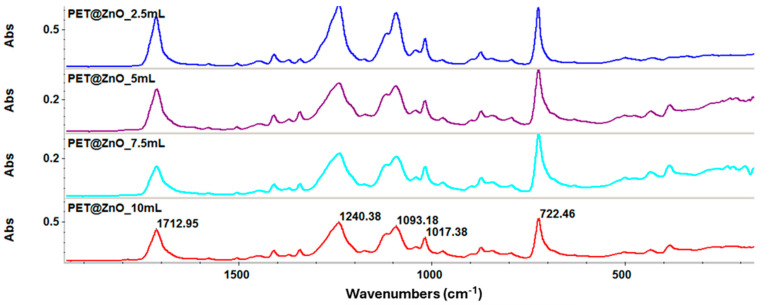
FT-IR spectra recorded for the PET@ZnO-type membranes.

The nanostructured membranes based on PET and ZnO nanoparticles were also characterized using Infrared Spectroscopy. The results obtained are presented in Figure 3. Absorption bands characteristic of PET are observed, as well as the characteristic absorption bands of the Zn-O bond at 436 cm^−1^ and 722 cm^−1^. At higher deposition rates, particularly 10 mL/h, this band becomes more intense, reflecting the increased incorporation of ZnO nanoparticles into the PET matrix. This correlates with the enhanced antimicrobial properties observed at this flow rate. Additionally, it is noted that the valorization of PET does not degrade its functional groups nor cause shifts in the absorption bands characteristic of the polymer. In more detail, the PET characteristic peaks at 1713 cm⁻^1^ (C=O stretching), 1240 cm⁻^1^ (C–O asymmetric stretching), and 1093 cm⁻^1^ (C–O symmetric stretching) remain unchanged [53,54], indicating that the deposition process does not affect the polymer’s intrinsic chemical structure. These results confirm the successful deposition of ZnO nanoparticles onto the PET fibers at all concentrations. Although FT-IR is limited in providing detailed information about nanoparticle interactions, it remains a valuable tool for confirming material composition.

While FT-IR analysis provided insight into ZnO’s structural and bonding characteristics on PET, no direct elemental analysis was conducted to quantify the amount of ZnO deposited. Future studies will include EDX analysis to provide more accurate quantification of ZnO content and better correlate the deposition rate with the amount of ZnO present.

**Figure 4:**
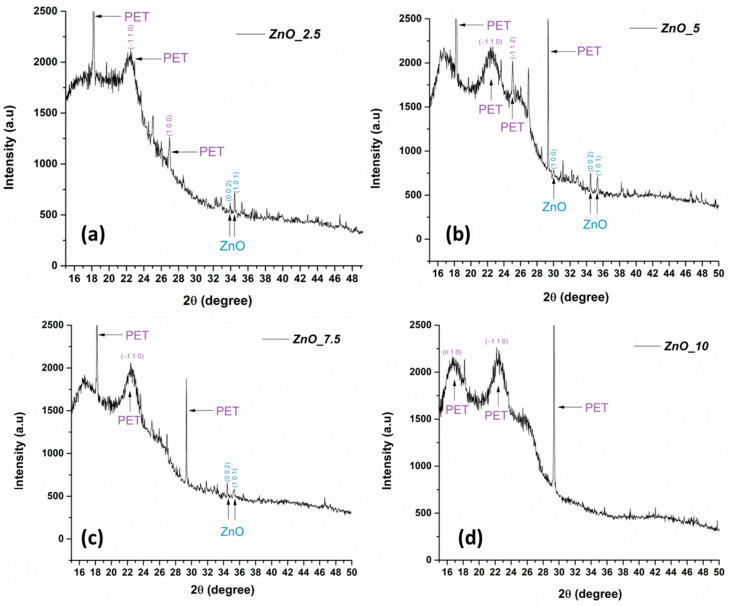
X-ray diffraction (XRD) patterns of PET@ZnO membranes, where (**a**) 2.5 mL/h, (**b**) 5 mL/h, (**c**) 7.5 mL/h, (**d**) 10 mL/h deposition rate.

The XRD patterns for the PET@ZnO membranes (Figure 4a–d) confirm the presence of both PET and ZnO phases. The characteristic diffraction peaks of PET correspond to the (0 1 0), (−1 1 0), and (1 0 0) planes, which are consistent with the crystalline structure of polyethylene terephthalate as reported in the literature [55]. The appearance of additional peaks at 2θ ≈ 30.1°, 33.9°, and 36.6° corresponds to the (1 0 0), (0 0 2), and (1 0 1) planes of ZnO, respectively, and matches the hexagonal wurtzite structure of ZnO [56]. Throughout all samples, the PET peaks remain visible, demonstrating that the crystalline structure of the PET substrate is preserved during the ZnO precipitation process. These observations align with the reported behavior of ZnO-coated polymer substrates in the literature, confirming the successful formation and stability of the PET@ZnO membranes.

Regarding thermal analysis (Table 1), the samples lose ~6% of the initial mass up to 340 °C. This process can be attributed to the elimination of some solvent/plasticizer molecules, as well as the partial oxidation of terminal moieties from the polymer chains. Also, in this interval, the melting endothermic effect is noticeable around 249–250 °C. The degradation of the polymer chains takes place in one step between 340–500 °C when most of the sample mass is lost. This process involves fragmentation of the polymer backbone and oxidation of the resulting fragments, as indicated by the multiple exothermic peaks, the most important being in the interval 460–470 °C when the residual carbonaceous mass is burned away [57]. After 500 °C, only a minor mass loss is recorded, due to the elimination of hydroxyl terminal moieties from the ZnO surface and densification of the inorganic oxide. The residual mass value for each sample (ZnO%) and the principal data from the thermal analysis are presented in Table 1.

### 3.2. Biological Characterizations

#### 3.2.1. Antimicrobial Effect

**Figure 5:**
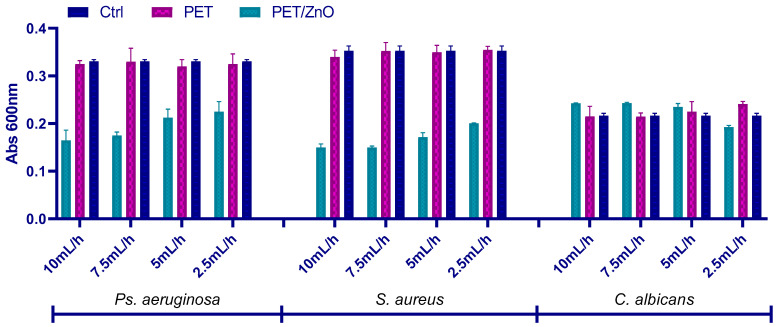
Absorbance values for *Ps. aeruginosa*, *S. aureus,* and *C. albicans* cultures, showing cell growth after 24 h in the presence of recycled PET@ZnO membranes.

The results demonstrate that the antimicrobial activity of the PET@ZnO membranes (Figure 5) is influenced by the ZnO content, which correlates with the deposition rate and findings from the thermogravimetric analysis (TGA). The TGA results (Table 1) confirmed an increase in residual ZnO content with higher deposition rates, ranging from 1.90% (PET@ZnO_2.5) to 8.95% (PET@ZnO_10).

For *S. aureus* cultures, the highest inhibition of planktonic growth was observed for PET@ZnO membranes prepared at 10 mL/h and 7.5 mL/h, which contain higher amounts of ZnO (8.95% and 6.10%, respectively). This suggests a direct correlation between the ZnO content and the antimicrobial efficacy, as the presence of ZnO nanoparticles enhances the material’s ability to disrupt bacterial growth. A similar trend was observed for *P. aeruginosa*, where PET@ZnO membranes exhibited greater inhibition of microbial growth compared to plain PET, with the inhibitory effect increasing proportionally to the ZnO content. However, the overall inhibition of *P. aeruginosa* was lower compared to *S. aureus*, possibly due to the increased resistance of Gram-negative bacteria to external stressors.

In the case of *C. albicans*, the antimicrobial effect was more uniform across all deposition rates. While the ZnO-containing membranes demonstrated slightly improved inhibitory effects compared to plain PET, the overall growth inhibition remained lower than for the bacterial strains. This result suggests that the antifungal efficacy of ZnO in the PET@ZnO membranes is less pronounced, potentially due to the differing structural and metabolic characteristics of yeast cells.

These observations confirm that the antimicrobial activity of PET@ZnO membranes is directly influenced by the ZnO content, as indicated by the TGA analysis, with higher ZnO concentrations leading to enhanced inhibition of microbial growth, particularly in bacterial strains.

**Figure 6:**
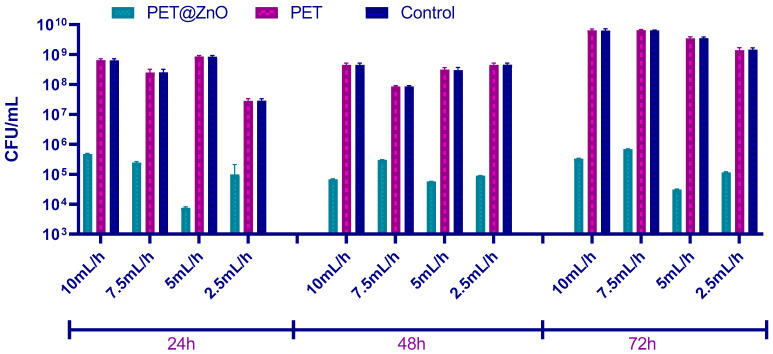
CFU/mL values showing the number of *S. aureus* cells in monospecific biofilms formed on the material surfaces after 24, 48, and 72 h at 37 °C.

**Figure 7:**
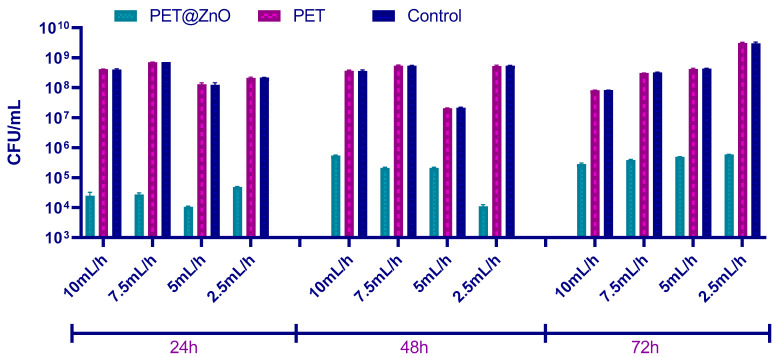
CFU/mL values showing the number of *Ps. aeruginosa* cells in monospecific biofilms formed on the material surfaces after 24, 48, and 72 h at 37 °C.

In the evaluation of biofilm formation, the results showed a significant reduction in the ability of *S. aureus* to form biofilms on the surfaces of PET membranes containing ZnO nanoparticles compared to plain PET and control samples (Figure 6). This inhibitory effect was consistent across all stages of biofilm development, including initial cell adhesion (24 h), biofilm maturation (48 h), and the dispersion phase (72 h), as illustrated in Figure 7. The PET@ZnO membranes demonstrated a substantial reduction in CFU/mL values at all time points, indicating their effectiveness in limiting bacterial colonization and biofilm growth.

At 24 h, fewer *S. aureus* cells adhered to the PET@ZnO surfaces, preventing the establishment of a dense biofilm. This effect persisted at 48 h, during the biofilm maturation phase, and remained significant at 72 h, when bacterial cells or aggregates typically disperse to colonize new surfaces. In contrast, the control and plain PET samples exhibited high CFU/mL values, supporting robust biofilm formation throughout the experiment. These results highlight the strong antimicrobial and anti-biofilm properties of PET membranes functionalized with ZnO nanoparticles.

*Pseudomonas aeruginosa* possesses numerous natural resistance mechanisms, making it a highly adaptable opportunistic pathogen capable of efficiently colonizing diverse environments. The biofilms formed by this bacterium are notoriously difficult to eliminate using conventional antimicrobial agents [58,59,60].

The results of this study demonstrate that the ability of this microorganism to form biofilms was significantly reduced on nanostructured PET materials containing ZnO. As shown in Figure 7, the PET@ZnO samples displayed a marked inhibitory effect across all three time intervals—24, 48, and 72 h—when compared to plain PET and control samples. At 24 h, the PET@ZnO membranes effectively limited initial biofilm formation, as indicated by the lower CFU/mL values. This inhibitory trend continued through the biofilm maturation phase (48 h) and the dispersion stage (72 h), where cell clusters typically detach to colonize new surfaces. In contrast, plain PET and control materials consistently supported extensive biofilm growth, as evidenced by the higher bacterial counts observed throughout the experiment.

**Figure 8:**
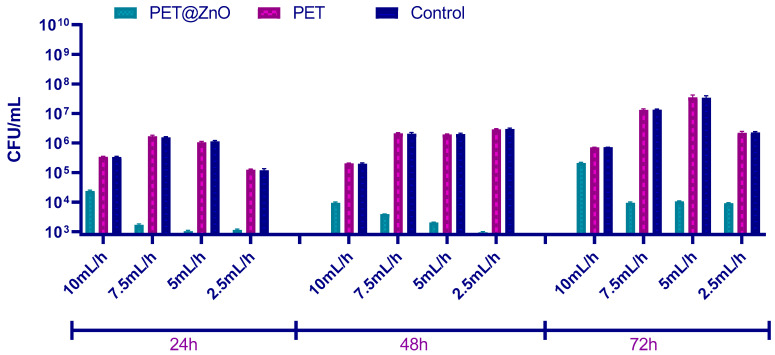
CFU/mL values showing the number of *C. albicans* cells in monospecific biofilms formed on the material surfaces after 24, 48, and 72 h at 37 °C.

The results obtained for *Candida albicans* biofilms demonstrate a significant inhibitory effect in the presence of PET@ZnO membranes compared to the control and plain PET samples. As shown in Figure 8, the CFU/mL values for biofilms formed on PET@ZnO surfaces were consistently lower at all analyzed time points (24, 48, and 72 h). This indicates that the PET@ZnO membranes effectively limit *C. albicans* biofilm formation despite the slight antifungal effects previously observed for planktonic *C. albicans* cultures.

The stronger inhibitory effect on *C. albicans* biofilms compared to planktonic cultures can be explained by the direct interaction between the ZnO nanoparticles and the biofilm matrix (Figure 8). The nanostructured surface likely disrupts initial adhesion and biofilm establishment, leading to reduced biofilm maturation and cell proliferation. However, the observed results must be considered in the context of fungal cell characteristics. Unlike bacterial cells, fungal cells possess a thicker and more complex cell wall composed of glucans, chitin, and mannoproteins, which offer greater protection and resistance to antimicrobial agents. Nevertheless, biofilm formation involves direct contact between the ZnO-containing fibers and the fungal biofilm, facilitating localized activity of ZnO nanoparticles and reactive oxygen species (ROS). This localized interaction disrupts the fungal biofilm structure, limiting its development over time.

While *C. albicans* biofilms are known for their robust extracellular polymeric substance (EPS) matrix, which can reduce nanoparticle penetration, the PET@ZnO materials appear to effectively inhibit biofilm formation through physical and chemical interactions. These findings emphasize the distinct response of *C. albicans* in biofilm form compared to planktonic cultures and demonstrate the promising ability of ZnO-functionalized PET membranes to mitigate fungal biofilm development.

**Figure 9:**
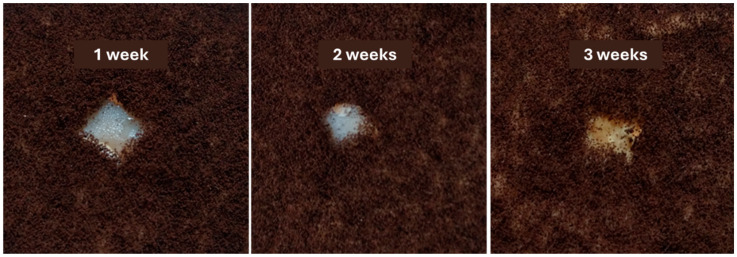
Appearance of *A. niger* cultures developed in the presence of the nanostructured PET@ZnO membranes, deposition rate 5 mL/h, over 1, 2, or 3 weeks.

The antifungal activity was evaluated using a strain of *Aspergillus niger*, cultivated for three weeks in the presence of the tested PET-based nanostructured membranes. The results demonstrated that the growth of the pathogenic microfungi was effectively inhibited both in the vicinity of, as well as beneath and over, the PET-based materials containing nanoparticles for the entire three-week period (Figure 9). 

#### 3.2.2. In Vitro Biocompatibility and Cytotoxic Profile

**Figure 10:**
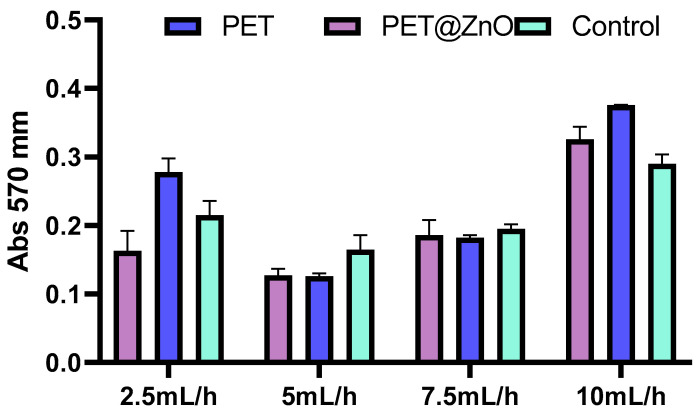
Graphical representation of the MTT technique results, represented by absorbance values at 570 nm, which suggest the optical density of the formazan released following the reduction reaction of the MTT reagent by the mitochondrial oxidoreductases of metabolically active cells in the presence of the tested PET materials.

The cytotoxicity of nanostructured membranes composed of recycled PET was evaluated using cultured human diploid cells. MTT assay results indicated that the activity of diploid cells in culture exhibited minor variations in response to the analyzed materials, which were influenced by the fiber deposition rate during electrospinning. In Figure 10, it can be observed that the metabolic activity and cell proliferation are relatively constant in the presence of nanostructured membranes based on PET, with disturbances in the sense of stimulated proliferation observed in the PET@ZnO samples at a 10 mL/h flow rate. This is indicative of enhanced cellular activity supported by the addition of ZnO into the polymer matrix. This increased activity suggests that the membranes may also support cellular proliferation, although further studies using methods such as BrdU or CFSE staining would be needed to confirm this directly.

Another method of cytotoxicity analysis is monitoring the induction of intracellular oxidative stress. The GSH method quantifies glutathione, a cellular antioxidant. Glutathione produced by cells is converted by glutathione S-transferase into its oxidized form. This transformation involves glutathione bound to a luciferin precursor being enzymatically converted into oxidized glutathione conjugated with luciferin, which emits light. The intensity of the emitted light correlates with the amount of glutathione transformed, reflecting the activity of glutathione S-transferase. A higher light intensity signifies greater glutathione synthesis, indicating increased cellular stress levels.

**Figure 11:**
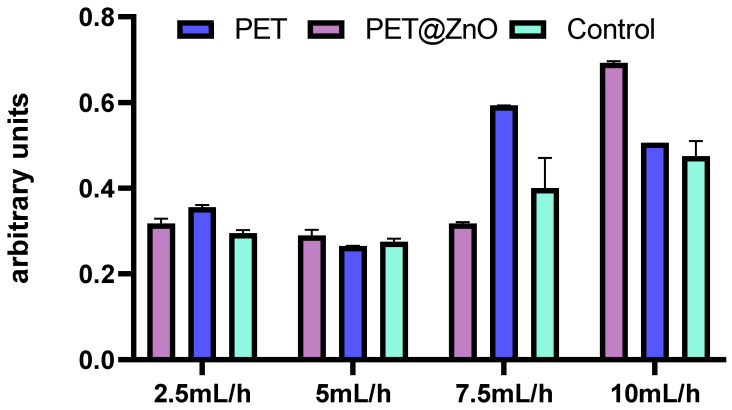
Luminescence values, expressed in arbitrary units, indicating glutathione S-transferase activity, which reflects oxidative stress levels in cultured diploid cells exposed to the obtained materials.

The results obtained by applying the GSH technique showed that the activity of glutathione S-transferase is altered in cells cultured in the presence of PET-control—flow rate 5 mL/h, PET-control—flow rate 7.5 mL/h, as well as PET@ZnO—flow rate 10 mL/h (Figure 11).

#### 3.2.3. In Vivo Results

**Figure 12:**
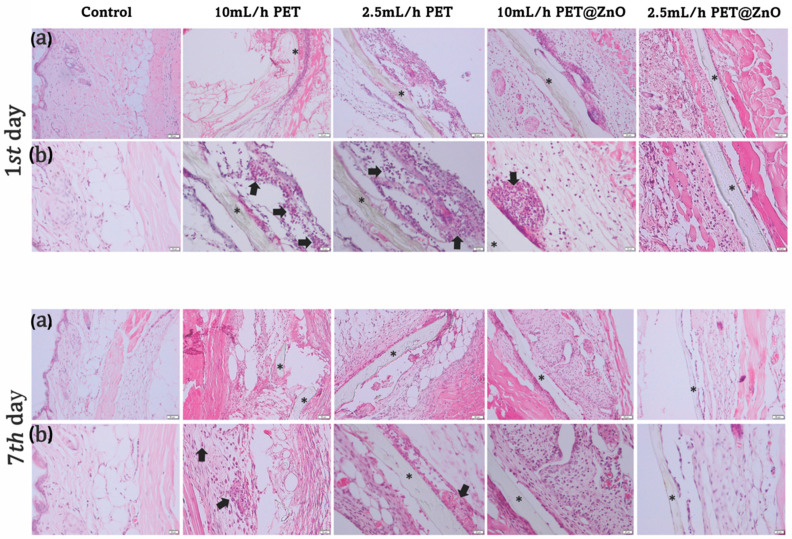
Histopathological analysis of PET@ZnO at 24 h and 7 days post-implantation in H&E stain. * Implanted material; arrow (**a**)—neutrophils; arrow (**b**)—macrophages; Scale bar 50 μm and 20 μm.

Daily post-implantation clinical analyses revealed no local or systemic side effects. Peri-implant edema was observed for PET control samples at 24 h post-implantation, persisting up to 7 days (Figure 12). This reaction increased with the rate of fiber deposition. At 24 h post-implantation, an inflammatory infiltrate, predominantly consisting of polymorphonuclear neutrophils (PMNs), was observed in the cutis and subcutis. They were replaced by macrophages at 7 days, which appeared predominantly in the granulation tissue surrounding the materials (Table 2).

For the PET@ZnO groups, inflammatory cells were mainly localized near the material.

**Figure 13:**
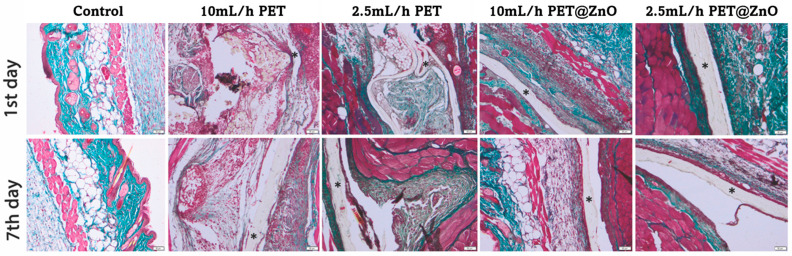
Collagen proliferation analysis after PET@ZnO subcutaneous implantation at 24 h and 14 days by Masson-Goldner trichrome stain; Scale bar 50 μm.

In vivo histological analysis at 7 days post-implantation showed marked collagen proliferation, particularly around the PET@ZnO membranes, promoting the formation of a well-defined fibrous capsule (Figure 13). ZnO-coated PET materials were surrounded by a clearly delimited area of granulation tissue consisting of fibroblasts, macrophages, and proliferating capillaries. These findings highlight ZnO’s role in supporting cellular growth and tissue regeneration.

## 4. Discussion

PET’s versatility and performance have created a substantial global demand for its utilization in a vast array of products, making it an indispensable material for numerous applications, including significant uses in beverage and food containers and synthetic fibers for clothing [61]. However, improper disposal and accumulation of PET waste have led to exacerbating pollution problems that require urgent management solutions [62,63,64]. For this reason, PET recycling is crucial for diminishing global pollution. Hence, understanding the properties and potential of this material is of great importance for finding ways to integrate recycled fibers and give them more meaningful purposes.

Numerous studies demonstrate that PET is a polyester of high importance for various industries, being a particularly feasible alternative material for biomedical applications [20,65,66]. PET-based materials can be employed in a plethora of biomedical devices, ranging from textiles [67], meshes [68,69], sutures [70,71], artificial ligaments [72], and grafts [71] to flexible biosensor substrates [73], and stents [74]. With such a wide range of possible uses, PET fibers made of recycled raw materials deserve special attention.

This study targeted the fabrication of PET-based membranes by electrospinning method coated with ZnO nanoparticles and evaluated their antimicrobial and antifungal properties and their possible application in medicine. Recognized for its versatility, cost-effectiveness, simplicity, and ability to generate polymeric fibers with small diameters, superior mechanical properties, and high surface area [75,76,77,78,79,80,81,82], electrospinning was rendered as a suitable technology for valorizing PET waste in our case. Specifically, the electrospinning technique allowed the fabrication of nanofibrous membranes formed of unordered fibers with diameters between 40 and 100 nm, on the surface of which granular ZnO nanostructures were attached. SEM and TEM investigations established that ZnO nanoparticles had sizes ranging from 10 to 20 nm and were non-uniformly distributed within the fibrous network. Both fiber sizes and nanoparticle dimensions are of the same order of magnitude as materials obtained by similar research studies involving electrospun functionalized structures, being at the inferior size limit of the ranges previously reported in the literature [45,47]. Moreover, ZnO nanoparticle size also agrees with our previous work that utilized the same synthesis method [44], demonstrating the repeatability and reproducibility of the process.

Electrospinning has also been a suitable processing method for maintaining the performance and characteristics of recycled PET materials. As revealed by FT-IR spectra, this valorization method does not degrade its functional groups nor cause shifts in the absorption bands characteristic of the polymer. FT-IR analysis has also allowed composition verification, confirming the presence of absorption bands characteristic of PET and ZnO in the investigated samples.

To establish the antimicrobial potential of the fabricated materials, relevant model microorganisms were chosen (i.e., Gram-positive bacteria—*S. aureus*, Gram-negative bacteria—*P. aeruginosa*; and yeasts—*C. albicans*) as both planktonic cultures and biofilms. These pathogens are among the most common infection threats in the clinical setting, having opportunistic behavior and increasing drug resistance [83,84]. Broadly, all types of investigated membranes were able to inhibit the growth and development of these harmful microorganisms in planktonic culture, with the highest activities being noted for higher fiber deposition rates. The samples containing ZnO nanoparticles had significantly higher inhibitory activity on free-floating microorganism cultures than pristine PET membranes, attributed to the intrinsic antimicrobial potential of these metal oxide nanostructures. The nanostructured PET-based membranes have also considerably reduced biofilm formation, with more significant inhibitory effects being noticed for PET@ZnO samples. In addition, the qualitative antifungal evaluation of PET-based materials with incorporated nanoparticles revealed that *A. niger* development was inhibited both in the vicinity and beneath or over these membranes.

ZnO nanoparticle concentration plays a critical role in the antimicrobial effectiveness of PET@ZnO membranes. Lower ZnO concentrations (2.5 mL/h) have less antimicrobial impact, though still significant when compared to plain PET membranes. At higher deposition rates, such as 7.5 mL/h and 10 mL/h, the increased ZnO concentration results in a more pronounced inhibition of both planktonic and biofilm-forming microorganisms, including *S. aureus* and *P. aeruginosa*. The enhanced antimicrobial activity can be attributed to the increased surface area of ZnO nanoparticles, which generates higher levels of reactive oxygen species (ROS) and leads to microbial membrane disruption [85,86,87]. Over time, the membranes demonstrated a consistent reduction in biofilm formation for *S. aureus* and *P. aeruginosa*, with the inhibition being more pronounced in the initial 24–48 h. This suggests ZnO’s sustained action against microbial colonization.

Similar studies have reported the antimicrobial properties of electrospun recycled PET functionalized with ZnO nanoparticles, particularly at higher ZnO concentrations, which enhance antibacterial and antifungal efficacy [47]. Our findings align with these reports, demonstrating that ZnO deposition rates significantly influence antimicrobial activity, with higher rates leading to improved microbial inhibition. Additionally, our study extends these findings by demonstrating the biocompatibility of PET@ZnO membranes and their capacity to promote collagen proliferation, highlighting their potential in tissue engineering and medical device applications.

Recent studies have also reported the utility of including ZnO nanoparticles for enhancing the antimicrobial potential of other electrospun polymeric fibers. Researchers have successfully tested these metal oxide-based nanostructures in combination with several natural and synthetic polymers, including cellulose acetate [88], gelatin [89], polycaprolactone [90], poly(methyl methacrylate) [91], polyvinyl alcohol [92,93], and polyacrylonitrile [94].

On the other hand, PET fibers have also been endowed with bactericidal and fungicidal activities by incorporating other nanoparticles to establish advanced antimicrobial solutions against clinically relevant pathogens. Combined with modified iron oxide nanoparticles [15] or with silver nanoparticles [14,95], recycled PET fibrillary membranes can be used as anti-infective tools against *S. aureus*, *P. aeruginosa*, and *C. albicans*.

Concerning the biocompatibility of the proposed materials, they exhibit no toxicity and do not interfere with the normal cell cycle progression of in vitro cultured diploid cells. Besides, no local or systemic side effects followed their implantation in model animals (i.e., CD1 mice). These findings agree with prior studies on PET-based nanofibrous materials [15,34,95,96,97], confirming the suitability of developed membranes for further biomedical uses.

The biocompatibility and antimicrobial properties of PET@ZnO membranes open up various possibilities for healthcare applications. These membranes can be utilized in antimicrobial wound dressings, preventing infection while promoting healing by supporting cell proliferation and collagen formation. Additionally, the anti-biofilm properties of these materials make them suitable for coating medical devices, reducing the risk of hospital-acquired infections. Furthermore, their potential in tissue engineering scaffolds offers a platform for both antimicrobial protection and enhanced cellular regeneration, addressing key challenges in wound healing and tissue repair.

## 5. Conclusions

This study highlights the successful development of electrospun membranes made of recycled PET nanofibers as a cost-effective valorization method for PET waste. Physicochemical characterizations revealed the synthesis of fibrillar membranes formed of unordered nanofibers (i.e., 40–100 nm in diameter) incorporated on their surface with ZnO nanoparticles of 10–20 nm. The PET-based nanostructured membranes demonstrated effective antimicrobial and anti-biofilm activity against a range of Gram-positive and Gram-negative bacteria, yeasts, and molds. Additionally, they are non-toxic and do not disrupt the normal cell cycle progression in diploid cells cultured in vitro. In vivo tests also revealed desirable outcomes, with no local or systemic side effects being observed throughout daily post-implantation clinical analyses.

Among the different fibers tested, the PET@ZnO membranes prepared with a solution flow rate of 10 mL/h demonstrated the most consistent fiber morphology, as observed through SEM analysis. These fibers exhibited a uniform distribution of ZnO nanoparticles across the surface, leading to enhanced antimicrobial activity compared to fibers prepared at lower flow rates. This uniformity in structure and nanoparticle coverage contributed to these membranes’ superior performance in antibacterial tests and cell viability assays. As a result, the fibers produced at 10 mL/h were considered the most promising for further development and applications.

The PET@ZnO membranes demonstrate a clear relationship between antioxidant properties, viability, and antibacterial activity. ZnO nanoparticles induce the formation of reactive oxygen species (ROS), which are effective in inhibiting bacterial growth. However, the antioxidant properties of the material play a crucial role in preventing ROS-induced damage to mammalian cells. Our findings suggest that the PET@ZnO membranes support cell viability by mitigating oxidative stress, thus balancing their antimicrobial efficacy with biocompatibility. Future work will include supplementary analyses to further validate these observations and explore concentration-dependent effects. Thus, the obtained results open up new perspectives for potential applications of recycled PET, particularly in the medical field. These include its use in combination with various inorganic nanostructures with antimicrobial and antibiofilm properties for advanced textiles, wound dressings, and surgical sutures. Such applications highlight the material’s versatility and potential to address critical needs in infection prevention.

## Figures and Tables

**Table 1:** Principal numeric data from thermal analysis.

Sample	Mass Loss RT-340 °C	Endo (°C)	Mass Loss 340–500 °C	Exo (°C)	Residual Mass (ZnO%)
PET_ZnO_2.5	6.04%	248.9 °C	90.28%	461.0 °C	1.90%
PET_ZnO_5	6.24%	249.0 °C	87.99%	465.0 °C	4.01%
PET_ZnO_7.5	6.45%	249.8 °C	85.70%	466.6 °C	6.10%
PET_ZnO_10	5.77%	250.1 °C	84.21%	466.8 °C	8.95%

**Table 2:** Histometric scoring of tissue reactions used to evaluate inflammation and neovascularization around subcutaneous implants.

Material	Implantation Period (Days)	Edema	PMN	M	E	RBC	F	NV
Control	1	-	-	-	-	-	-	-
	7	-	-	-	-	-	-	-
PET 10 mL/h	1	++++	++++	+	-	+	-	-
	7	++++	+	++++	-	+	+++	-
PET 2.5 mL/h	1	++	+++	+	-	+	-	-
	7	+	+	+++	-	+	++	+
PET@ZnO 10 mL/h	1	+	++	+	-	-	+	-
	7	-	+	++	-	-	++++	+
PET@ZnO 2.5 mL/h	1	-	+	+	-	-	+	-
	7	-	-	+	-	+	+++	++

PMN: Polymorphonuclear neutrophils; M: Macrophages; E: Eosinophils; RBC: Extravasated red blood cells; F: Fibroblasts; NV: Neovascularization; Tissue reactions are rated from - (not present) to ++++ (extensive).

## Data Availability

The raw data supporting the conclusions of this article will be made available by the authors upon request.
